# Entering the ‘big data’ era in medicinal chemistry: molecular promiscuity analysis revisited

**DOI:** 10.4155/fsoa-2017-0001

**Published:** 2017-03-06

**Authors:** Ye Hu, Jürgen Bajorath

**Affiliations:** 1 Department of Life Science Informatics, B-IT, LIMES Program Unit Chemical Biology & Medicinal Chemistry, Rheinische Friedrich-Wilhelms-Universität, Dahlmannstr. 2, D-53113 Bonn, Germany

**Keywords:** big data concept, big data criteria, compound activity data, computational mining, drug discovery, medicinal chemistry, polypharmacology, promiscuity

## Abstract

The ‘big data’ concept plays an increasingly important role in many scientific fields. Big data involves more than unprecedentedly large volumes of data that become available. Different criteria characterizing big data must be carefully considered in computational data mining, as we discuss herein focusing on medicinal chemistry. This is a scientific discipline where big data is beginning to emerge and provide new opportunities. For example, the ability of many drugs to specifically interact with multiple targets, termed promiscuity, forms the molecular basis of polypharmacology, a hot topic in drug discovery. Compound promiscuity analysis is an area that is much influenced by big data phenomena. Different results are obtained depending on chosen data selection and confidence criteria, as we also demonstrate.

## Lay abstract

‘Big data’ is affecting many areas of life, more so than we might realize. It is often not well understood what big data really mean. This is also true in science. For example, medicinal chemistry, which is a conservative scientific discipline and slow to respond to new trends, is currently entering the big data era. This provides many new opportunities and challenges, as exemplified by the computational study of biological activities of drugs and other compounds from medicinal chemistry.

For the anticancer drug imatinib (top), a kinase inhibitor, the histogram indicates the relative number of target annotations on the basis of low- (red), medium- (green) or high-confidence (blue) activity data. Hence, the magnitude of detectable promiscuity (multitarget activity) decreases with increasing data confidence.

## Graphical abstract

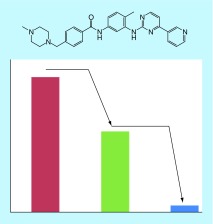

The electronic data deluge and ‘big data’ phenomena affect essentially all areas of life [1]. What is ‘big data’? In 2001, industry analyst D Laney associated ‘big data’ with the need to control the volume, velocity and variety of data [2]. Volume, velocity and variety represent the often-cited ‘3Vs’ of big data, which then became an integral part of the general definition put forward by Gartner, Inc., a large international information technology firm [3]:

“Big data is high-volume, high-velocity and/or high-variety information assets that demand cost-effective, innovative forms of information processing that enable enhanced insight, decision making, and process automation.”

On the basis of this definition, a characteristic feature of big data is that conventional data processing, storage and transfer infrastructures are insufficient for handling such data. Moreover, big data might be structured to varying degrees or even be completely unstructured. Importantly, big data characteristics go much beyond the technical level and scientific big data criteria must be carefully considered when drawing conclusions from data analysis.

The fundamental big data characterization of Laney has been further extended over the years in various ways and the ‘3Vs’ have recently become ‘7Vs’ with M van Rijmenam adding veracity, variability, visualization and value [4]. This is probably not the end of it – and there is of course also no reason to limit big data attributes to ‘Vs’. However, core of big data issues are well accounted for by the original attributes and a few others.

In science, big data has been on the agenda for at least a decade, in some fields more so than in others. In particle physics, colliders produce massive amounts of data, and this already for more than a decade [5]. In biology, the big data wave hits more recently, due to the advent of efficient genomics technologies providing high throughput [6]. Consequently, genomic sequencing data began to grow exponentially in 2008 [6]. Thus, in biology, big data is no news and building and evolving computational infrastructures for handling and analyzing big data, especially genomic sequencing data, still is a major topic in bioinformatics. In physics and biology, big data is measured in ‘petabytes’ (1 petabyte = 10^15^ bytes) and cloud computing has become an indispensable part of big data storage and analysis.

Big data originating from biology has also entered drug discovery [7,8], where it is complemented with, for example, data from high-throughput screens, array experiments, imaging, pharmacology and clinical investigations, which – at least in part – also have big data characteristics by now. These developments considerably challenge drug discovery environments and result in the need to explore new training concepts for discovery scientists, further emphasize interdisciplinary approaches, and add data science to the spectrum of drug discovery-relevant disciplines [8].

Compared to the situation in biology, big data is still in its infancy in medicinal chemistry, which is another pillar of drug discovery. Clearly, the variety of data associated with cells and organisms is principally much larger than of data associated with chemical compounds – and so are the ensuing data volumes that can be generated. However, although big data trends are only beginning to emerge in medicinal chemistry [9,10], it is evident that this field will also be increasingly influenced by big data issues. For example, proprietary medicinal chemistry projects in the pharmaceutical industry will inevitably need to take compound activity data into consideration that is rapidly accumulating in the public domain [9]. Merging internal and external data and viewing chemical optimization of compound series in an overarching context represents a departure from the long-established operating culture of medicinal chemistry and presents new challenges to practicing chemists. However, the opportunities provided by extracting knowledge from rapidly growing amounts of compounds and publicly available activity data cannot be disregarded.

An important point to emphasize is that our discussion of big data issues in medicinal chemistry primarily focuses on experimental data, which typically require computational analysis – but not on computationally generated data. If we take into consideration that literally thousands of chemical descriptors and properties can be calculated for any given compound, it is evident that the amount of compound-associated data can be further increased by orders of magnitude through computational chemistry. However, such ‘theoretical’ big data represents another category to which big data criteria considered herein only vaguely apply and the utility of which – and relevance for the practice of medicinal chemistry – might also be questioned.

Big data in medicinal chemistry has also been associated with specific attributes. While we have stressed complexity, heterogeneity and confidence as additional big data criteria of particular relevance for medicinal chemistry [9], for reasons detailed below, Lusher *et al*. have promoted ‘5Vs’ for medicinal chemistry by adding veracity and value to the original ‘3Vs’ [10]. Data complexity, heterogeneity and confidence are related to some of the ‘Vs’ but are distinct from them. For example, data variability influences complexity, heterogeneity and confidence all of which, however, include additional parameters, as further discussed below. On the other hand, veracity, the ‘truth’ of data, can be called into question at varying confidence levels (and is often – but not always – also related to variability). One might add that the value of data is mostly relevant for commercial enterprises and as such – albeit justifiable – not uniformly applicable to the big data concept.

Regardless of the origins of compound activity data, how can one make use of these data and learn from it? For example, structure-activity relationships (SARs) can be extracted on a large-scale from compounds active against current pharmaceutical targets and used to complement chemical optimization efforts. Moreover, it is also possible to systematically explore multitarget activities of small molecules on the basis of available activity data. Such multitarget activities provide the foundation of polypharmacology [11–15], an emerging concept in drug discovery, according to which many bioactive compounds and drugs elicit their physiological and therapeutic effects through interactions with multiple – rather than single – targets. The ability of small molecules to specifically interact with multiple targets is also rationalized as promiscuity [16], which needs to be clearly distinguished from nonspecific interactions and assay artifacts caused by aggregating molecules [17,18] or (reactive) interference compounds [19,20].

Another point of consideration is that promiscuity analysis typically does not take relative compound potencies into account. Even weak activities should be considered, which could give rise to side effects. Therefore, promiscuity must be differentiated from selectivity. A promiscuous compound might not be equally potent against all of its targets. Rather, it might display higher potency against one or more targets over others. Accordingly, a promiscuous compound might or might not display target selectivity. Interestingly, however, the majority of promiscuous compounds we have identified through data mining were found to display comparably high potency against their targets [21], whereas promiscuous compounds that were selective for a primary target over others (on the basis of high vs low potency values) were less frequently observed [21].

Furthermore, in the context of promiscuity analysis, the terms ‘frequent hitter’ [22] and ‘privileged (sub)structure’ [23], which are commonly used in medicinal chemistry, might also be considered. Frequent hitters refer to compounds that are often active in biological screens, which might either be due to promiscuity, as rationalized herein, or undesired artifacts. Privileged structures refer to common core structures of compounds that are primarily active against a given target family over others, but not specific for individual members of the family, which would result in intrafamily promiscuity.

Exploring compound promiscuity as the molecular basis of polypharmacology represents an interesting application for emerging big data in medicinal chemistry. This is the case because views concerning the potential magnitude of promiscuity and polypharmacology widely differ in the field and are often articulated on the basis of intuition or subjective expectations [15], without carefully considering available activity data [24]. With the advent of big data in medicinal chemistry, opportunities increase to arrive at quantitative estimates of promiscuity that are backed up by statistically significant data volumes.

This contribution focuses on emerging big data issues in medicinal chemistry and shows how big data criteria influence the analysis of compound activity data. Primarily conceived as a perspective-type article, it was designed to be ‘data-driven,’ consistent with its major theme. Accordingly, we intentionally combine the discussion of big data aspects, exemplary studies and personal viewpoints with a large-scale analysis of compound activity data, resulting in a perspective with research components. For reasons discussed above, promiscuity analysis was chosen as an exemplary topic for compound data mining to monitor potential progression of multitarget activities, as data volumes massively grow, and re-evaluate previous promiscuity estimates in a rigorous manner.

## Materials & methods

### Databases

Three public domain databases were used as data sources including ChEMBL (release 22; ChEMBL22) [25,26], the major repository for compounds and activity data from medicinal chemistry literature and patent sources, PubChem BioAssay [27], the major repository for screening data, and DrugBank (version 5.0.3) [28], a comprehensive source of approved and investigational drugs.

### Compound structures

The structures of all qualifying compounds from different data sources were standardized using the OpenEye OEChem toolkit [29]. Canonical simplified molecular-input line-entry system (SMILES) strings [30] were generated to map compounds across different databases.

### Targets

ChEMBL provides two internal numerical index systems as target identifiers including ‘tid’ and ‘CHEMBL_ID’. Targets from PubChem are designated using gene identifiers. For target annotations in DrugBank, UniProt IDs [31] are given. To standardize targets from different sources and map them, UniProt IDs were chosen as the target identifiers. Thus, target designations in ChEMBL and PubChem were converted to UniProt IDs (if available) using the ID mapping function of ChEMBL or UniProt.

### Matched molecular pairs

Structural relationships between compounds were systematically explored by applying the matched molecular pair (MMP) formalism [32]. An MMP is generally defined as a pair of compounds that only differ by a chemical change at a single site [32], in other words, the exchange of a pair of substructures, termed a ‘chemical transformation’ [33]. Previously defined transformation size restrictions were applied to confine MMPs to pairs of structural analogs [34]. MMPs were calculated using an in-house version of a fragmentation algorithm [33] utilizing the OpenEye toolkit [29].

### Compound promiscuity

For each compound, its ‘promiscuity degree’ (PD) was defined as the number of targets a compound is known to be active against. It is calculated as the total number of target annotations available at a given point in time. Compounds for which only a single target was available (PD 1) were classified as target specific. In addition, for compounds from ChEMBL and drugs mapped to ChEMBL, the progression of PDs was monitored over time from 1995 to 2015 on the basis of ChEMBL activity records and publication dates extracted from them.

## Compound activity data volumes

Big data phenomena are often primarily associated with unprecedentedly large volumes of available data, which is an oversimplification. In medicinal chemistry, these data mostly consist of compound structures and biological activity records. Pharmacology data or clinical records are only infrequently available, at least in the public domain. For structures of small molecules, the ZINC database [35] serves as a good example. ZINC collects compounds that are relevant for medicinal chemistry from vendor sources as well as other databases. The current ZINC 15 release (November 2015) reports approximately 220 million compounds with a molecular weight of up to 1000 Da, more than 120 million of which are proposed to be purchasable from medicinal chemistry vendors worldwide [35]. By contrast, the former release of ZINC 14 (August 2015) contained 23 million of compounds, in other words, an order of magnitude less. This example alone mirrors the emerging big data trend in medicinal chemistry. However, ZINC compounds are not associated with activity data. Thus, going beyond molecular structures, other public databases must be taken into consideration, in particular, ChEMBL and PubChem, the currently most important repositories for compounds and activity from medicinal chemistry and biological screening, respectively. Since ChEMBL also imports data from PubChem, the boundaries between these databases become rather fluid, but the data origins are recorded and can be easily traced. As reported in Table 1, ChEMBL22 contains nearly 1.7 million compounds from medicinal chemistry sources that are active against more than 11,200 targets, forming a total of more than 14 million ligand–target interactions. In the practice of medicinal chemistry, such numbers would have been considered science fiction just a few years ago. ChEMBL22 also incorporates activity data from PubChem. The PubChem BioAssay database comprises more than 1.2 million assays with nearly 2.3 million tested compounds, yielding a total of more than 230 million activity annotations covering more than 10,000 target proteins. As reported in Table 1, a subset of 437,257 screening compounds was tested in both primary and confirmatory assays (with varying compound concentrations and determination of IC_50_ values) that involved 456 and 596 targets, respectively. In primary assay, these compounds yielded a total of nearly 1 million activity annotations and in confirmatory assay, more than 500,000 annotations. In addition to these large volumes of compounds and activity data from medicinal chemistry and screening, DrugBank 5.0.3 currently reports 1564 approved small-molecule drugs annotated with 1836 targets forming a total of 11,387 drug–target interactions, which provides a basis for systematically comparing activity annotations of drugs and other bioactive compounds at earlier stages of the drug development process.

**Table 1.** **Volumes of currently available compound activity data.**

DrugBank 5.0.3	#Approved drugs	1564		
	#Targets	1836		
	#Drug–target interactions	11,387		
ChEMBL22 (all)	#Compounds	1,686,695		
	#Targets	11,224		
	#Compound–target interactions	14,371,197		
		K_i_	IC_50_	K_i_+IC_50_
ChEMBL22 (high-confidence)	#Compounds	67,179	168,559	222,431
	#Targets	867	1578	1686
	#Compound–target interactions	111,466	231,189	327,660
		Primary		Confirmatory
PubChem BioAssay	#Compounds	437,257		
	#Active compounds	267,418		196,607
	#Targets	456		596
	#Compound–target interactions	922,306		507,321

Reported are currently available compound activity data from different sources including DrugBank 5.0.3, ChEMBL22 and the PubChem BioAssay database. For ChEMBL22, high-confidence activity data subsets and corresponding target annotations are reported separately for K_i_ and IC_50_ measurements as well as combined K_i_ and IC_50_ data. For PubChem BioAssay, compounds tested in both primary and confirmatory assays are given. For each assay category, the number of active compounds and target annotations is separately provided.

## Rationalizing big data

Importantly, focusing on rapidly growing data volumes alone provides an incomplete picture of big data, as already implied by the relevance of the ‘3Vs’ [2]. This also applies to big data in medicinal chemistry and drug discovery. In our view, extracting knowledge and learning from big data are especially challenged by increasing data complexity and heterogeneity across different sources. Moreover, data confidence issues critically influence the results of data mining efforts and conclusions drawn from them. In our view, data complexity, heterogeneity and confidence represent prime big data criteria that must be carefully considered in data analysis and knowledge extraction. Hence, the following discussion will focus on these issues.

## Data confidence levels

**Figure 1.**
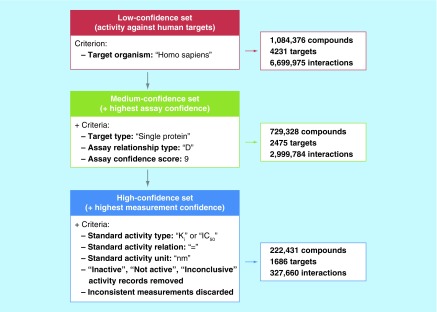
**Datasets with varying confidence levels.** The selection of low-, medium- and high-confidence sets is schematically illustrated. For each set, selection criteria applied for retrieving corresponding bioactivity records from ChEMBL are listed (left) and the numbers of qualifying compounds, targets and compound-target interactions are given (right).

Figure 1 summarizes criteria for the assembly of compound datasets at varying confidence levels and lists corresponding settings of selection parameters that are available in ChEMBL. Applying these settings, ChEMBL datasets reported herein can be fully reproduced.

In our studies, we generally distinguish between low-, medium- and high-confidence compound activity data. Low-confidence data are obtained by collecting all available activity for a given target, regardless of assay or activity measurement criteria. As reported in Figure 1, ChEMBL22 contains more than a million compounds that are active against 4231 human targets, corresponding to a total of nearly 6.7 million compound–target interactions. For human targets, this represents the low-confidence dataset, taking all available activity annotations into account. Medium-confidence activity data are obtained by selecting well-defined assays that assess direct interactions of compounds with single protein targets at the highest level of assay confidence (i.e., confidence score 9, following the ChEMBL assay classification scheme). This reduces the number of active compounds from about a million to approximately 730,000 and the total number of ligand–target interactions from approximately 6.7 to approximately 3 million (Figure 1). Finally, the high-confidence dataset is obtained by requiring not only highest possible assay confidence but also highest measurement confidence. This is achieved by limiting activity measurements to explicitly specified (assay-dependent) IC_50_ values and (assay-independent) equilibrium constants (K_i_ values) and eliminating activity records with inconsistent or inconclusive information (Figure 1). These specifications significantly reduce the number of ChEMBL compounds active against human targets when proceeding from medium- to high-confidence data. In the latter dataset, approximately 222,000 compounds remain that are active against 1686 targets, forming a total of nearly 328,000 interactions. Because IC_50_ and K_i_ values can in principle not be directly compared, an additional high-confidence data criterion can be applied by separating qualifying compounds into different subsets on the basis of these measurement types. As reported in Table 1, the K_i_ subset of ChEMBL22 comprises 67,179 compounds that are active against 867 targets and the IC_50_ subset 168,559 compounds active against 1578 targets (compounds for which both K_i_ and IC_50_ values are available occur in both subsets). We emphasize that even at the highest level of activity data confidence, ChEMBL22 yields nearly 328,000 ligand–target interactions, which provide a sound basis for exploring SARs on a large scale and determining compound PDs.

## Assessing compound promiscuity

Multitarget activities of small molecules provide the basis of polypharmacology, which is frequently implicated in efficacy [36], but are also responsible for unwanted side effects of drugs or candidate compounds [13]. In addition, exploring multitarget activities provides the basis for drug repurposing [37,38], i.e., for finding new therapeutic applications for approved drugs. Accordingly, quantifying and better understanding compound promiscuity is of considerable interest for drug discovery research. For the further analysis and discussion of PDs, we distinguish between drugs and other compounds that are active against therapeutically relevant targets but that are not drugs (termed ‘bioactive compounds’).

### Computational infrastructures

Prior to focusing on quantitative assessments of promiscuity of drugs and bioactive compounds through data mining, we briefly review previous studies that have reported computational tools and data structures for promiscuity analysis or other large-scale data mining analyses. For example, the ChEMBLSpace graphical explorer, a standalone Java implementation, was developed to generate and visualize compound-based target networks on the basis of ChEMBL activity records and identify compounds with sought after multitarget activity profiles [39]. An interactive Web-based analysis tool termed PharmaTrek was introduced to mine multitarget activity space by defining, for example, a pool of targets and potency thresholds of compounds active against these targets [40]. Accordingly, PharmaTrek was applied to ChEMBL to visualize compound activity profiles. Another Web-based resource was reported to extract compound activity counts from PubChem assays and calculate various compound descriptors for subsequent analysis [41]. Furthermore, for migrating and analyzing PubChem assay data, the BioAssay Research Database structure was generated that also included a number of data mining and visualization tools [42]. Moreover, for drugs, the PROMISCUOUS database was introduced comprising 25,000 entries also including experimental drugs and drugs withdrawn from the market. In addition, 21,500 drug–target interactions and 104,000 drug-based protein–protein relationships were compiled from public resources via text and data mining and manually curated [43]. Target and side effect profiles of drugs were then visualized in network representations that captured relationships between drugs and target proteins and between drugs and side effects. The computational tools described above aid in compound data and information extraction from major repositories.

**Promiscuity estimates**

Other investigations have attempted to quantitatively estimate the degree of promiscuity of drugs and bioactive compounds. For drugs, network representations of drug–target interactions have played an important role in early attempts to assess promiscuity [44,45]. From different databases, drugs, targets and interactions were collected and analyzed in drug–target networks. From such data representations, it was estimated that a drug might on average most likely interact with six different targets [44,45]. Depending on the datasets that were used, interactions per drug ranged from about 3 to 13 [45]. For drugs directed against different major therapeutic target families, estimates were further refined, resulting in two to eight targets per drug, depending on the particular family [14]. Comparable estimates were obtained when approved and experimental drugs taken from DrugBank were mapped back to ChEMBL and drug promiscuity was monitored over a time period of 15 years on the basis of high-confidence activity data [46]. However, for bioactive compounds, a different picture emerged. In an early analysis of PubChem, multitarget activities were analyzed on the basis of more than 600 assays [47]. It was found that approximately 58% of the screening compounds had only single-target activity in combined primary and confirmatory assays. When only confirmatory assays were considered, approximately 74% of the compounds displayed single-target activity [47]. Moreover, on the basis of high-confidence activity data extracted from ChEMBL, it was determined that an active compound interacted on average with only one to two targets, with no significant variations across different therapeutic target families [48]. When compound promiscuity was monitored over time on the basis of ChEMBL activity records dating back to the mid-1970s, increases in average promiscuity beyond the level of two targets per compound were only observed when medium- or low-confidence activity data were considered [49]. For high-confidence data, average PDs remained constant at about 1.5 targets per compound, even during times of exponential data growth beginning in 2010 [49]. Hence, for bioactive compounds, lower PDs than for drugs were consistently determined.

It should be noted that ChEMBL does not contain records of inactivity or assay frequency of compounds, which are generally not provided in the medicinal chemistry literature upon which ChEMBL is based. Thus, on the basis of ChEMBL activity records, it cannot be determined how often and against how many targets compounds might have been tested. This situation gives rise to an occasionally voiced critique concerning PDs derived from ChEMBL (or proprietary compound databases). Essentially, as long as not all available compounds have been tested against all possible targets, promiscuity estimates are likely to be lower than ‘true’ PDs, due to data incompleteness, which also affected the generation of drug–target networks [44,45]. Notably, obtaining a complete compound–target interaction matrix represents the ultimate goal of chemogenomics [50], which will most likely remain elusive from a practical viewpoint. Thus, data incompleteness will continue to affect promiscuity analysis; the key question is to what extent? Clearly, given the advent of big data in medicinal chemistry, current activity data volumes are already so large – as discussed earlier – that it must be possible to extract statistically meaningful trends from these data. Without doubt, estimates quantifying what currently available data tell us are scientifically more valid than subjective expectation values or partly informed guesses.

Importantly, data incompleteness can be balanced for large subsets of active compounds. For example, different from ChEMBL records, assay frequency information for active compounds can be extracted from PubChem, enabling the identification of most extensively tested screening hits. More than 430,000 compounds were identified that were on average tested in more than 450 primary and confirmatory assays and their PDs were determined [51]. Even for these most extensively assayed small molecules, mean PDs were only slightly higher than observed for ChEMBL compounds, with 2.5 targets per screening hit [51], thus lending further credence to promiscuity estimates for bioactive compounds.

As a prominent example, ATP site-directed kinase inhibitors used for cancer therapy have become a paradigm for promiscuous compounds [52,53]. However, this compound class also serves as an instructive example for potential ambiguities in judging about promiscuity. Although ATP site-directed kinase inhibitors are often thought to display a high degree of promiscuity, given the largely conserved ATP binding site in many kinases, analysis of high-confidence activity data according to Figure 1 does not reveal generally higher promiscuity than of compounds directed against other major classes of therapeutic targets [48,54]. Data sparseness may certainly influence this assessment, but there are other – perhaps more critical – factors to consider. Importantly, many practicing drug discovery scientists judge activities of kinase inhibitors – as well as of compounds and drugs directed against other targets – on the basis of profiling [55] or small-molecule array experiments [56], which are nowadays widely carried out in the pharmaceutical industry, rather than on the basis of global data views. However, under the experimental conditions of profiling or array experiments, unusually high degrees of compound promiscuity are frequently observed [57] resulting from, for example, the use of high compound concentrations and/or solid-phase assays. Thus, multitarget activities observed in such cases must be considered with caution in light of the specific experimental conditions applied and are difficult to generalize. Exclusively basing judgment on a given high-throughput assay format might well lead to biased views of promiscuity and other compound properties, which also recommends complementing in-house results with external data. Hence, striving for a balanced assessment of results obtained under specific experimental conditions in light of already available internal or external data is – in our view – a *conditio sine qua non* for sound science including research in drug discovery environments. However, in our experience, there often remains considerable room for improvement in evaluating data from diverse sources in context.

## Promiscuity analysis in light of big data criteria

In the following, we present up-to-date results of promiscuity analysis paying close attention to big data criteria discussed above, thus highlighting a number of critical issues.

### Data growth

**Figure 2.**
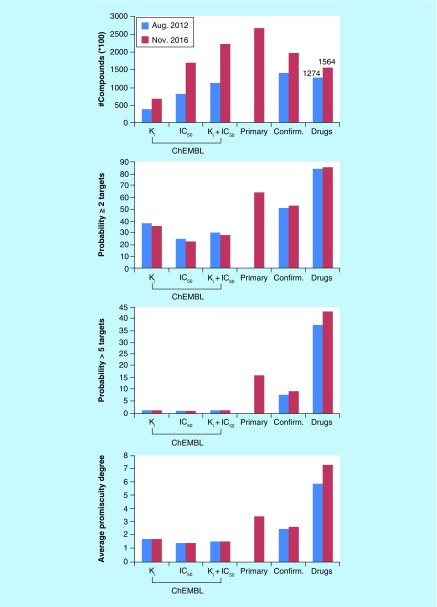

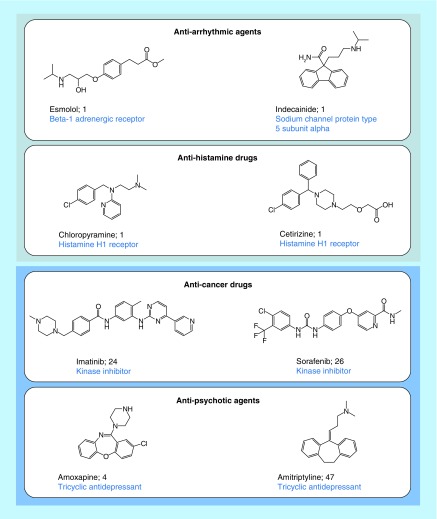
**Data volumes versus compound promiscuity.** In **(A)**, the number of compounds available from different sources in August 2012 [48] and November 2016 and their calculated promiscuity degrees are compared. Sources include ChEMBL, primary assays (first considered in 2016) and confirmatory assays from PubChem, and approved drugs from DrugBank. In the top panel, the *y*-axis is scaled by 100 for compounds from ChEMBL and PubChem (but not drugs, as reported in the figure). In the middle panels, the probability of compounds from different sources to interact with at least 2 or more than 5 targets is reported, respectively. In the bottom panel, the average promiscuity degree of compounds from different sources is compared. In **(B)**, 4 exemplary drugs annotated with single targets and four promiscuous drugs are shown. For each drug, its name and the number of target annotations available in DrugBank 5.0.3 are reported. For single-target drugs, the target name is provided and for promiscuous drugs, a primary application or functional annotation is given.

The top chart in Figure 2A monitors the growth of compound activity between August 2012, when our previous promiscuity analysis was carried out [48], and November 2016. High-confidence activity data from ChEMBL22 were taken into consideration together with PubChem primary assays (first considered in 2016) and confirmatory assays and approved drugs from DrugBank 5.0.3. Approximately 56,000 additional compounds were tested in confirmatory assays over the past 4 years, whereas the number of compounds in the ChEMBL-derived high-confidence set essentially doubled, which was mostly due to large increases in the number of compounds with available IC_50_ measurements. Thus, there was massive growth in bioactive compounds with high-confidence activity data. Furthermore, as one might expect, the number of approved drugs only increased moderately by 290 drugs over the 4-year period.

### Promiscuity degrees

The bottom graph in Figure 2A compares PDs of bioactive compounds, screening compounds and drugs in 2012 and 2016. Although the number of bioactive compounds with high-confidence activity data doubled within 4 years, the average PD remained constant at about 1.5 (K_i_ + IC_50_). For 2016, an average PD of 3.4 was obtained for PubChem compounds from primary assays (initial screening hits). For compounds from confirmatory assays, the PD also remained essentially constant at 2.5. Thus, for bioactive compounds including confirmed screening hits, detectable promiscuity continued to be low, although many more compounds became available. By contrast, for drugs, there was a notable further increase in average drug promiscuity from 5.9 to 7.3. Thus, the PD of drugs was much higher than of bioactive compounds including primary screening hits. In the two middle graphs of Figure 2A, the probability is reported for compounds to be active against at least two or more than five targets, respectively. These probabilities were calculated from the global distributions of PDs and also remained largely constant over time. For a bioactive compound with high-confidence activity data, the probability to be active against at least 2 targets was approximately 30%, for a compound from confirmatory assays approximately 50% and for an approved drug approximately 85%. By contrast, for bioactive compounds, the probability to be active against more than 5 targets was less than 1%. For PubChem compounds from primary and confirmatory assays, these probabilities were approximately 16% and less than 10%, respectively. Furthermore, the proportion of drugs with activity against at least 2 targets was comparable between 2012 and 2016 (i.e., 84.1 vs 85.6%). However, there still was a greater than 40% probability of drugs to interact with more than 5 targets in 2016, with an increase from 37.4 to 43.1%. Thus, drugs with higher degrees of promiscuity (>5 targets) were more frequent in 2016 than in 2012.

### Rationale for observed promiscuity levels?

The results in Figure 2A clearly show that the promiscuity of bioactive compounds is generally low and constant over time while data volumes massively grow and drugs are much more promiscuous than other bioactive compounds. Can we rationalize these observations? Several points may be considered. It is certainly possible – and also likely – that many bioactive compounds from medicinal chemistry have only been tested against limited numbers of targets at the time of their publication. In this context, we also note that the majority of single – as well as multitarget compounds available in ChEMBL can be traced back to single-source publications, rather than multiple publications [58]. Thus, it may be the case that many compounds are not extensively retested once they are published. On the other hand, negative data and inactive compounds are generally not reported in the medicinal chemistry literature and, therefore, assay frequency remains unknown. At the same time, profiling experiments and selectivity screens are frequently published. Moreover, most extensively assayed screening hits matched the low to average PDs for compounds from PubChem confirmatory assays [51]. Hence, the situation is multifactorial and complex. Regardless of any speculations about the origins and magnitude of promiscuity among bioactive compounds, what can be concluded with certainty is that PDs of bioactive compounds have remained overall low while the number of compounds with available high-confidence activity data has doubled over the past 4 years. Thus, these findings are based on unprecedentedly large volumes of data from medicinal chemistry. Furthermore, there also is no doubt that approved drugs are on average much more promiscuous than other bioactive compounds. It is of course very likely that drug candidates and drugs are more extensively tested than other bioactive compounds. However, it is also possible that subsets of promiscuous drugs might preferentially be selected for efficacy during clinical evaluation, depending on the therapeutic applications. On the basis of currently available data, not all drugs are promiscuous, which would also be difficult to reconcile, given that the quest for target-specific or -selective compounds has dominated drug discovery efforts over the past 2 to 3 decades. Figure 2B shows exemplary drugs for different therapeutic applications that are only annotated with a single target or highly promiscuous. The comparison emphasizes high PDs of individual kinase inhibitors used for cancer treatment or ligands of G protein-coupled receptors used as antipsychotic agents. In fact, a close look at the drug data in Figure 2A suggests that confined subsets of highly promiscuous drugs might strongly influence average PDs. This possibility was further investigated by recalculating drug PDs following iterative removal of increasing numbers of highly promiscuous drugs. As reported in Table 2, when the 50 most promiscuous drugs were removed, the mean and median degrees of drug promiscuity decreased from 7.3 to 6.4 and 5.0 to 4.5, respectively. When the 100 most promicuous drugs were removed, a further decrease of mean and median PDs to 5.8 and 4.0 was observed, respectively. These 100 drugs were reported to interact with 22–85 targets and their average PD was 29.6. Removal of the 200 most promiscuous drugs only led to a further reduction of the mean PD to 4.9 while the median degree of 4.0 remained constant. These 200 drugs were on average active against 23.7 targets, ranging from 15 to 85 targets. Thus, subsets of highly promiscuous drugs had a notable influence on global promiscuity assessment. However, even after removal of the 200 most promiscuous drugs (i.e., ∼13% of all approved drugs), mean and median PDs were still significantly higher than those determined for ChEMBL and PubChem compounds.

**Table 2.** **Promiscuity after iterative removal of highly promiscuous drugs.**

**Removal of top N most promiscuous drugs**	**Promiscuity degree**	
	**Average**	**Median**
0	7.3	5.0
50	6.4	4.5
80	6.0	4.0
100	5.8	4.0
150	5.3	4.0
200	4.9	4.0

Average and median drug promiscuity degrees of drugs from DrugBank 5.0.3 are reported after removal of the 50, 80, 100, 150 or 200 most promiscuous drugs, respectively.

### Data heterogeneity

**Figure 3.**
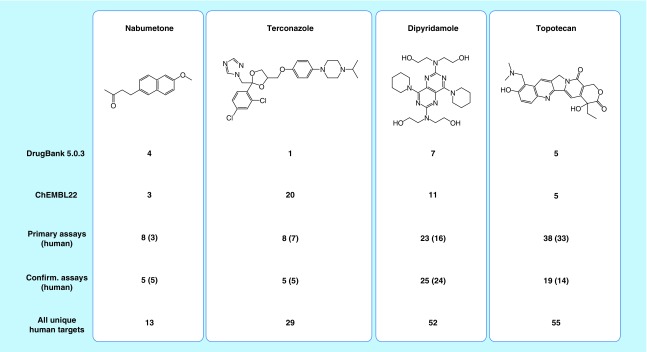
**Data heterogeneity.** For four drugs, the number of target annotations from different sources is given. ChEMBL22 refers to its combined high-confidence set (K_i_ + IC_50_). The number of all unique human targets was obtained by combining target annotations from different sources.

In addition to data volumes, heterogeneity of data across different sources was discussed above as another big data criterion. Figure 3 compares target annotations of four exemplary drugs available in different databases and highlights the relevance of the heterogeneity criterion. For nabumetone and topotecan, DrugBank and ChEMBL report very similar or the same number of targets (maximum five), whereas PubChem assays indicate much larger numbers of targets for topotecan, yielding a total of 55 (!) unique human targets, but not for nabumetone. A similar picture is observed for dipyridamole that is, however, annotated with 7 targets in DrugBank and 11 in ChEMBL. By contrast, terconazole has only 1 target in DrugBank but 20 in ChEMBL and a total of 29 unique human targets. These representative examples illustrate that there often is a high level of inconsistency among target annotations for drugs and other bioactive compounds across different data sources, which we have observed many times. At face value, completely different conclusions would be drawn concerning the PDs and target profiles of the well characterized compounds shown in Figure 3, depending on the databases used, if one was unaware of these heterogeneity issues. These comparisons also emphasize the need for stringent and consistently applied data selection criteria and protocols for across-database analyses.

### Data complexity

**Figure 4.**
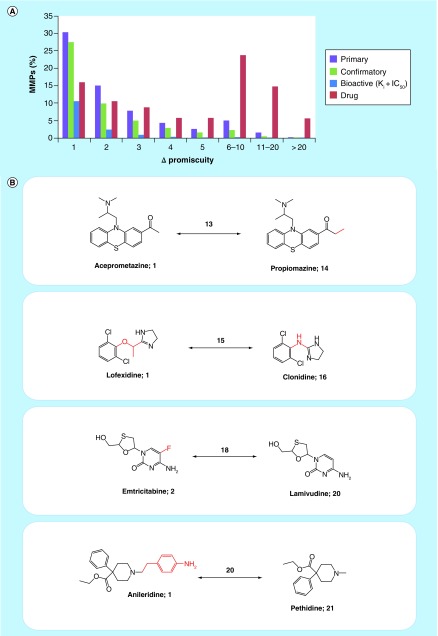
**Structural relationships versus promiscuity degrees.** **(A)** Shows the proportions of MMPs from different sources encoding increasing differences in PDs (ΔPromiscuity). **(B)** Shows four exemplary pairs of drugs that formed MMPs and had large differences in promiscuity. For each drug, the number of targets reported in DrugBank 5.0.3 is given and pairwise differences are reported above the arrows. Structural modifications distinguishing MMP partners are highlighted in red. MMP: Matched molecular pair.

Complexity of compound activity data, another big data criterion we have emphasized, is reflected at different levels. This begins with the formal composition of activity records, which varies considerably across different repositories and might also include ontologies and pharmacological information [59], and further extends to scientific issues. An excellent example for high scientific complexity of compound data is provided by analyzing the interplay between structural relationships and PDs. Structural relationships between active compounds were systematically determined by MMP search calculations. Each detected MMP represented a pair of structural analogs that were only distinguished by a confined chemical change at a single site. Figure 4A shows the proportion of MMPs from primary and confirmatory PubChem assay compounds, the high-confidence ChEMBL set (bioactive), and drugs that encode increasing promiscuity differences. As one might anticipate, many structural analogs were active against very similar numbers of targets. In fact, for ChEMBL compounds with available high-confidence activity data, pairs of structural analogs forming approximately 87% of the MMPs were active against the same number of targets (ΔPromiscuity = 0; not shown) and the proportion of MMPs encoding increasing promiscuity differences greater than 1 rapidly declined. MMPs of assay hits more frequently encoded larger differences in promiscuity. However, a striking finding was that significant proportions of drug MMPs encoded large differences in promiscuity. For example, more than 22% of the drug MMPs consisted of structural analogs with differences in PDs of 6–10 and approximately 13% with differences of 11–20. Figure 4B shows exemplary pairs of structurally analogous drugs with large differences in promiscuity. These pairs are reminiscent of ‘promiscuity cliffs,’ originally defined as structural analogs with large differences in promiscuity that originated from compound array experiments [57]. For screening compounds from array experiments, such differences might be attributed to specific experimental conditions or artifacts, but for approved drugs, this would hardly be possible. For example, aceprometazine and propiomazine in Figure 4B only differ by a methyl group, a minute structural modification, while the former drug is annotated with a single target and the latter with 14 targets. Similarly, emtricitabine (2 targets) and lamivudine (20 targets) only differ by single fluorine ring substituent. Such structure–promiscuity relationships among drugs are puzzling and have not been reported before. However, together with similar relationships recently detected for other bioactive compounds [60], they are a part of the emerging big data spectrum in medicinal chemistry and drug discovery. As to whether small-structural modifications may indeed cause such significant differences in promiscuity remain to be determined. Other possible reasons for these observations are far from being obvious. Hence, there is much room for future research to further explore the molecular and/or data basis of complex promiscuity relationships among drugs and other bioactive compounds, the origins of which are currently only very little understood.

### Varying data confidence levels

**Figure 5.**
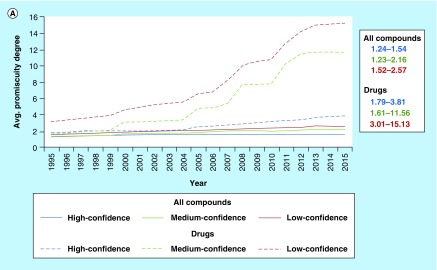

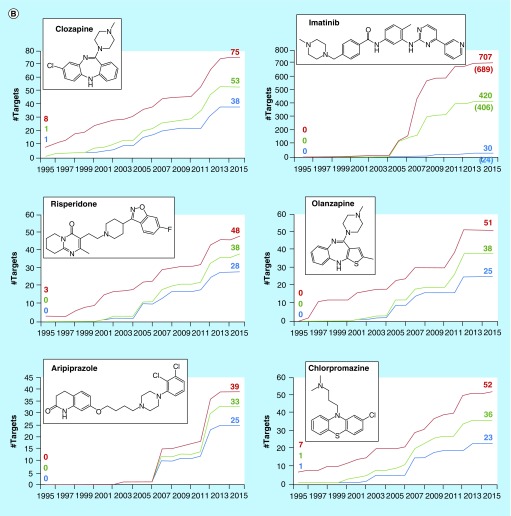
**Progression of compound and drug promiscuity over time.** In **(A)**, the progression of average promiscuity degrees over time is shown for all bioactive compounds (solid lines) and drugs (dashed lines) from the low- (red), medium- (green) and combined high-confidence (blue) datasets of ChEMBL22 and mean PD ranges are reported (right). **(B)** Shows six drugs from the high-confidence dataset with largest PD increases. For imatinib, the number of target annotations at different confidence levels available in ChEMBL18 [46] is given in parentheses.

The critically important role of confidence criteria for big data analysis is illustrated by following the progression of compound promiscuity over time. To these ends, drugs were mapped to ChEMBL and activity records were selected on the basis of different confidence criteria according to Figure 1 and organized by publication dates. Figure 5A shows how average PDs have evolved over two decades (1995–2015) for drugs and other bioactive compounds on the basis of low-, medium- and high-confidence data. For bioactive compounds, mean PDs were found to increase over time from about 1.2 (high- and medium-confidence) and 1.5 (low-) to about 1.5 (high-), 2.2 (medium-) and 2.6 (low-confidence). Thus, for high-confidence data, the increase in mean promiscuity of bioactive compounds was only very small during times of large-magnitude data growth. Furthermore, there was a clearly detectable but only moderate relative increase in promiscuity with decreasing data confidence. By contrast, for drugs, data confidence-dependent differences in promiscuity progression were in part unexpectedly large. On the basis of high-confidence data, drug PDs increased from about 1.8 to 3.8, which was still comparable in magnitude to bioactive compounds with low-confidence data. However, for drugs from the medium- and low-confidence sets, average promiscuity increased from about 1.6 and 3.0 to 11.6 and 15.1, respectively. Thus, completely different views of drug promiscuity and its progression would be obtained depending on the chosen data confidence levels, with an in part astonishing relative increase in (apparent) promiscuity with decreasing data confidence. Clearly, unawareness of such data confidence issues and their consequences will render data analysis questionable at best. Figure 5B shows examples of drugs with largest increases in PDs over time on the basis of high-confidence activity data and illustrates that promiscuity estimates at lower confidence levels become unrealistic. All of these drugs were highly promiscuous on the basis of cumulative activity records in 2015. The single drug with the largest PD increase over time – from 1 to 38 – was clozapine, a G protein-coupled receptor ligand and antipsychotic agent used since the early 1970s. Clozapine was followed by imatinib, with an increase in its PD from 0 to 30. Imatinib was developed during the early 2000s and became the first ATP-site-directed kinase inhibitor marketed for cancer therapy (Gleevec). For clozapine, high-, medium- and low-confidence data yielded final PDs of 38, 53 and 75, respectively. Thus, its PD essentially doubled for low- compared to high-confidence data. For imatinib, however, the PD for high-, medium- and low-confidence data increased from 30, which might be realistic in magnitude for a highly promiscuous kinase inhibitor, to 420 and 707, respectively. In the imatinib graph, numbers in parentheses report the corresponding values for 2012, confirming a further increase in PDs during the past 4 years. Clearly, for medium- and low-confidence data (i.e., all available activity annotations), estimates for imatinib become completely unrealistic, also taking into consideration that the human kinome comprises 518 different kinases [61]. It is self-evident that a compound with activity against 700 or so targets, if it would exist, could never be used therapeutically; considering any form of ‘specific’ ligand–target interactions would be absurd at this level. Thus, the imatinib example alone teaches us important lessons about the essential role of confidence criteria in big data analysis.

### Assay frequency

**Figure 6.**
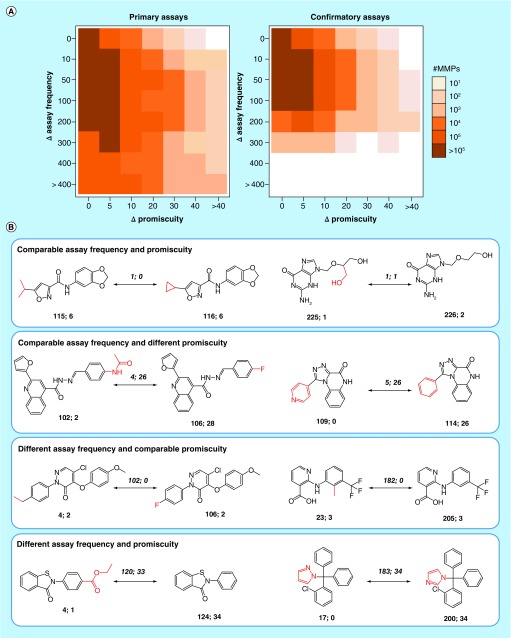
**Assay frequency versus promiscuity degrees.** **(A)** Shows heat maps capturing differences in assay frequencies and promiscuity degrees for MMP-forming hits from primary (left) and confirmatory (right) PubChem assays. Cells represent MMPs having a given difference in assay frequency (ΔAssay frequency) and promiscuity degree (ΔPromiscuity) and are color-coded by MMP population density as indicated. **(B)** Shows MMPs representing different relationships between assay frequency and promiscuity degrees. For each compound, the number of confirmatory assays and targets is reported. For example, ‘115; 6’ means that the compound was tested in 115 confirmatory assays and found to be active against 6 targets. For MMP partners, the differences in assay frequency and PD are given in italics above the arrows and structural modifications are highlighted in red. MMP: Matched molecular pair.

Finally, we focus the analysis on assay frequency, which – as discussed above – is often a missing variable in promiscuity assessment, depending on the data sources. Therefore, for all MMPs formed by PubChem compounds from primary and confirmatory assays, respectively, encoded PD differences (according to Figure 4A) were systematically related to differences in assay frequency between MMP partners. The results are shown in Figure 6A. The heat map representations reveal that there was no evident relationship between differences in PDs and assay frequency of structural analogs forming MMPs. Rather, differences in promiscuity were more or less evenly distributed over the entire range of observed differences in assay frequency. Overall largest MMP population density occurred in sections of assay frequency differences of up to 200 assays and accompanying PD differences of up to 5. All possible relationships between assay frequency and PDs were observed, as illustrated in Figure 6B. These relationships included comparable confirmatory assay frequency and comparable or significantly different PDs of structural analogs (top two panels) and different assay frequency and comparable or different PDs (bottom two panels). Figure 6B shows representative examples. The second panel includes two close structural analogs, only distinguished by a heteroatom substitution in a ring, which were tested in 109 and 114 confirmatory assays, respectively. The first compound was found to be consistently inactive in all assays it was evaluated in, whereas the second was active against 26 different targets. Furthermore, the third panel includes an exemplary pair of structural analogs that were found to be active against the same three targets while one was tested in 23 and the other in 205 different confirmatory assays. Thus, a variety of relationships were observed and increasing assay frequency did not correlate with increasing PDs. These findings further complemented the far from being simplistic picture of compound and drug promiscuity derived from emerging big data.

## Conclusion

Medicinal chemistry is experiencing the advent of the big data era, which biology already entered more than a decade ago, due to the availability of high-throughput genomics technologies. In medicinal chemistry, which is an integral part of drug discovery and traditionally a conservative scientific discipline, big data primarily comprise rapidly increasing numbers of compounds and volumes of associated activity data. Herein, we have discussed important big data criteria including data heterogeneity, complexity and confidence that – in addition to mere data volumes – play a decisive role in utilizing big data and learning from it. The computational analysis of promiscuity of drugs and other bioactive compounds as the molecular basis of polypharmacology was selected as an instructive example of how big data influences and challenges research in medicinal chemistry.

## Future perspective

While the practice of medicinal chemistry is just beginning to experience big data phenomena, it is evident that big data will play an increasingly important role going forward. More awareness of big data issues and potential caveats will still need to be raised to positively impact the field. For example, albeit rather obvious, opportunities provided by complementing in-house chemical optimization efforts with public domain SAR data are not yet widely appreciated, although they should be routinely considered. Incorporating external data – and knowledge derived from such data – into discovery projects and decision-making challenges the practice of medicinal chemistry. Big data characteristics further complicate matters. Promiscuity analysis serves as a good example. Currently, multitarget activities of small molecules and their potential utility or drawbacks for drug discovery are still only little understood. In particular, judging about the magnitude of promiscuity among drugs and candidate compounds is often much more subject to speculation and subjective views than rigorous scientific assessment. Albeit difficult to analyze, big data provides an attractive opportunity for a more detailed scientific assessment of molecular promiscuity in drug discovery. As we have demonstrated herein, the scenario is far from being simple and rigorous data analysis, carefully taking big data criteria into consideration, provides as many new questions as insights. What we can firmly conclude on the basis of currently available compound activity data is that approved drugs are on average much more promiscuous than other bioactive compounds and bioactive compounds are overall less promiscuous than often assumed. Any other conclusions or expectations are not backed up by reliable data and belong to the realm of speculation. From careful data mining and analysis, new questions arise. As we have shown, a variety of structure–promiscuity relationships can be observed. Are these relationships ‘real’ or largely determined by experimental parameters and the way data are generated? Furthermore, might it ultimately be possible to learn the molecular language that promiscuous compounds and targets use in communicating with each other and prospectively design compounds with desired multitarget activities? If so, how might polypharmacology-driven efficacy of drugs and unwanted side effects be balanced? Clearly, tackling big compound data and learning from these data will also provide starting points for deriving new experimentally testable hypotheses with potential for exciting future research, which – after all – is good news for science.

**Executive summary**

- ‘Big data’ plays a role in many scientific fields and areas of life.
- The big data concept involves more than mere data volumes, which is often not well understood.
- Compared to the situation in biology, big data is still in its infancy in medicinal chemistry, but can no longer be ignored.
- In addition to volume, velocity and variety, which are often used to characterize big data, we have focused on *heterogeneity*, *complexity* and *confidence* as additional big data criteria for medicinal chemistry and beyond.
- The analysis of compound promiscuity as the molecular basis of polypharmacology is much influenced by big data phenomena.
- Rigorous large-scale analysis of compound activity data from different sources indicates that drugs are generally much more promiscuous than other bioactive compounds, which – on the other hand – are less promiscuous than often thought.

## References

[1] Mayer-Schönberger V, Cukier K (2013). *Big data: A Revolution That Will Transform How We Live, Work, And Think*.

[2] 3D data management: controlling data volume, velocity, and variety. http://www.blogs.gartner.com/doug-laney/files/2012/01/ad949%E2%80%933D-Data-Management-Controlling-Data-Volume-Velocity-and-Variety.pdf.

[3] Gartner http://www.gartner.com/it-glossary/big-data/.

[4] Datafloq Why the 3Vs are not sufficient to describe big data. http://datafloq.com/read/3vs-sufficient-describe-big-data/166/.

[5] Lynch C (2008). Big data: how do your data grow?. *Nature*.

[6] Marx V (2013). The big challenges of big data. *Nature*.

[7] Al-Lazikani B, Workman P (2016). Minimizing bias in target selection by exploiting multidisciplinary Big Data and the protein interactome. *Future Med. Chem.*.

[8] Bajorath J, Jenkins J, Overington J, Walters WP (2016). Drug discovery and development in the era of big data. *Future Med. Chem.*.

[9] Hu Y, Bajorath J (2014). Learning from ‘big data’: compounds and targets. *Drug Discov. Today*.

[10] Lusher SJ, McGuire R, van Schaik RC, Nicholson CD, de Vlieg J (2014). Data-driven medicinal chemistry in the era of big data. *Drug Discovery Today*.

[11] Paolini GV, Shapland RHB, van Hoorn WP, Mason JS, Hopkins AL (2006). Global mapping of pharmacological space. *Nat. Biotechnol.*.

[12] Hopkins AL (2008). Network pharmacology: the next paradigm in drug discovery. *Nat. Chem. Biol.*.

[13] Boran AD, Iyengar R (2010). Systems approaches to polypharmacology and drug discovery. *Curr. Opin. Drug Discov. Devel.*.

[14] Jalencas X, Mestres J (2013). On the origins of drug polypharmacology. *Med. Chem. Comm.*.

[15] Anighoro A, Bajorath J, Rastelli G (2014). Polypharmacology: challenges and opportunities in drug discovery: miniperspective. *J. Med. Chem.*.

[16] Hu Y, Bajorath J (2013). Compound promiscuity - what can we learn from current data. *Drug Discov. Today*.

[17] McGovern SL, Caselli E, Grigorieff NA (1996). Common mechanism underlying promiscuous inhibitors from virtual and high-throughput screening. *J. Med. Chem.*.

[18] Shoichet BK (2006). Screening in a spirit haunted world. *Drug Discov. Today*.

[19] Baell JB, Holloway GA (2010). New substructure filters for removal of pan assay interference compounds (PAINS) from screening libraries and for their exclusion in bioassays. *J. Med. Chem.*.

[20] Baell J, Walters MA (2014). Chemistry: chemical con artists foil drug discovery. *Nature*.

[21] Hu Y, Bajorath J (2013). Promiscuity profiles of bioactive compounds: potency range and difference distributions and the relation to target numbers and families. *Med. Chem. Commun.*.

[22] Schneider G, Neidhart W, Giller T, Schmid G (1999). “Scaffold-hopping” by topological pharmacophore search: a contribution to virtual screening. *Angew. Chem. Int. Ed. Engl.*.

[23] Müller G (2003). Medicinal chemistry of target family-directed masterkeys. *Drug Discov. Today*.

[24] Hu Y, Bajorath J (2013). How promiscuous are pharmaceutically relevant compounds? A data-driven assessment. *AAPS J.*.

[25] Gaulton A, Bellis LJ, Bento AP (2012). ChEMBL: a large-scale bioactivity database for drug discovery. *Nucleic Acids Res.*.

[26] Gaulton A, Hersey A, Nowotka M (2017). The ChEMBL database in 2017. *Nucleic Acids Res.*.

[27] Wang Y, Xiao J, Suzek TO (2012). PubChem's BioAssay database. *Nucleic Acids Res.*.

[28] Law V, Knox C, Djoumbou Y (2014). DrugBank 4.0: shedding new light on drug metabolism. *Nucleic Acids Res.*.

[29] OEChem TK (2012). http://www.eyesopen.com/.

[30] Weininger D (1988). SMILES, a chemical language and information system. 1. Introduction to methodology and encoding rules. *J. Chem. Inf. Comput. Sci.*.

[31] UniProt Consortium (2010). The Universal Protein Resource (UniProt) in 2010. *Nucleic Acids Res.*.

[32] Kenny PW, Sadowski J, Oprea TI (2004). Structure modification in chemical databases. *Chemoinformatics in Drug Discovery*.

[33] Hussain J, Rea C (2010). Computationally efficient algorithm to identify matched molecular pairs (MMPs) in large data sets. *J. Chem. Inf. Model.*.

[34] Hu X, Hu Y, Vogt M, Stumpfe D, Bajorath J (2012). MMP-cliffs: systematic identification of activity cliffs on the basis of matched molecular pairs. *J. Chem. Inf. Model.*.

[35] Sterling T, Irwin JJ (2015). ZINC 15 – ligand discovery for everyone. *J. Chem. Inf. Model.*.

[36] Reddy AS, Zhang S (2013). Polypharmacology: drug discovery for the future. *Expert Rev. Clin. Pharmacol.*.

[37] Ashburn TT, Thor KB (2004). Drug repositioning: identifying and developing new uses for existing drugs. *Nat. Rev. Drug Discov.*.

[38] Chong CR, Sullivan DJ (2007). New uses for old drugs. *Nature*.

[39] Fechner N, Papadatos G, Evans D (2013). ChEMBLSpace – a graphical explorer of the chemogenomic space covered by the ChEMBL database. *Bioinformatics*.

[40] Carrascosa MC, Massaguer OL, Mestres J (2012). PharmaTrek: a semantic web explorer for open innovation in multitarget drug discovery. *Mol. Inform.*.

[41] Canny SA, Cruz Y, Southern MR, Griffin PR (2012). PubChem promiscuity: a web resource for gathering compound promiscuity data from PubChem. *Bioinformatics*.

[42] Howe EA, de Souza A, Lahr DL (2015). BioAssay Research Database (BARD), chemical biology and probe-development enabled by structured metadata and result types. *Nucleic Acids Res.*.

[43] von Eichborn J, Murgueitio MS, Dunkel M, Koerner S, Bourne PE, Preissner R (2011). PROMISCUOUS: a database for network-based drug-repositioning. *Nucleic Acids Res.*.

[44] Mestres J, Gregori-Puigjané E, Valverde S, Solé RV (2008). Data completeness – the Achilles heel of drug-target networks. *Nat. Biotechnol.*.

[45] Mestres J, Gregori-Puigjané E, Valverde S, Solé RV (2009). The topology of drug-target interaction networks: implicit dependence on drug properties and target families. *Mol. Biosyst.*.

[46] Hu Y, Bajorath J (2014). Monitoring drug promiscuity over time. *F1000Research*.

[47] Han L, Wang Y, Bryant SH (2009). A survey of across-target bioactivity results of small molecules in PubChem. *Bioinformatics*.

[48] Hu Y, Bajorath J (2013). High-resolution view of compound promiscuity. *F1000Research*.

[49] Hu Y, Jasial S, Bajorath J (2015). Promiscuity progression of bioactive compounds over time. *F1000Research*.

[50] Bredel M, Jacoby E (2004). Chemogenomics: an emerging strategy for rapid target and drug discovery. *Nat. Rev. Genet.*.

[51] Jasial S, Hu Y, Bajorath J (2016). Determining the degree of promiscuity of extensively assayed compounds. *PLoS ONE*.

[52] Knight ZA, Lin H, Shokat KM (2010). Targeting the cancer kinome through polypharmacology. *Nat. Rev. Cancer*.

[53] Morphy R (2009). Selectively nonselective kinase inhibition: striking the right balance. *J. Med. Chem.*.

[54] Hu Y, Furtmann N, Bajorath J (2015). Current compound coverage of the kinome. *J. Med. Chem.*.

[55] Duffner JL, Clemons PA, Koehler AN (2007). A pipeline for ligand discovery using small-molecule microarrays. *Curr. Opin. Chem. Biol.*.

[56] Goldstein DM, Gray NS, Zarrinkar PP (2008). High-throughput kinase profiling as a platform for drug discovery. *Nat. Rev. Drug Discov.*.

[57] Dimova D, Hu Y, Bajorath J (2012). Matched molecular pair analysis of small molecule microarray data identifies promiscuity cliffs and reveals molecular origins of extreme compound promiscuity. *J. Med. Chem.*.

[58] Hu Y, Bajorath J (2016). Analyzing compound activity records and promiscuity degrees in light of publication statistics. *F1000Research*.

[59] Williams AJ, Harland L, Groth P (2012). Open PHACTS: semantic interoperability for drug discovery. *Drug Discov. Today*.

[60] Dimova D, Gilberg E, Bajorath J (2017). Identification and analysis of promiscuity cliffs formed by bioactive compounds and experimental implications. *RSC Adv.*.

[61] Manning G, Whyte DB, Martinez R, Hunter T, Sudarsanam S (2002). The protein kinase complement of the human genome. *Science*.

